# *CUL3*-related neurodevelopmental disorder: Clinical phenotype of 20 new individuals and identification of a potential phenotype-associated episignature

**DOI:** 10.1016/j.xhgg.2024.100380

**Published:** 2024-11-04

**Authors:** Liselot van der Laan, Ananília Silva, Lotte Kleinendorst, Kathleen Rooney, Sadegheh Haghshenas, Peter Lauffer, Yasemin Alanay, Pratibha Bhai, Alfredo Brusco, Sonja de Munnik, Bert B.A. de Vries, Angelica Delgado Vega, Marc Engelen, Johanna C. Herkert, Ron Hochstenbach, Saskia Hopman, Sarina G. Kant, Ryutaro Kira, Mitsuhiro Kato, Boris Keren, Hester Y. Kroes, Michael A. Levy, Ngu Lock-Hock, Saskia M. Maas, Grazia M.S. Mancini, Carlo Marcelis, Naomichi Matsumoto, Takeshi Mizuguchi, Alessandro Mussa, Cyril Mignot, Anu Närhi, Ann Nordgren, Rolph Pfundt, Abeltje M. Polstra, Slavica Trajkova, Yolande van Bever, Marie José van den Boogaard, Jasper J. van der Smagt, Tahsin Stefan Barakat, Mariëlle Alders, Marcel M.A.M. Mannens, Bekim Sadikovic, Mieke M. van Haelst, Peter Henneman

**Affiliations:** 1 Amsterdam UMC, Department of Human Genetics, University of Amsterdam, Amsterdam, the Netherlands; 2 Amsterdam Reproduction & Development Research Institute, Amsterdam, the Netherlands; 3 Department of Pathology and Laboratory Medicine, Western University, London, ON, Canada; 4 Verspeeten Clinical Genome Centre, London Health Science Centre, London, ON, Canada; 5 Division of Pediatric Genetics, Department of Pediatrics, Acibadem University, School of Medicine, Istanbul, Turkey; 6 Rare Diseases and Orphan Drugs Application and Research Center-ACURARE, Acibadem University, Istanbul, Turkey; 7 Department of Medical Sciences, University of Torino, Torino, Italy; 8 Medical Genetics Unit, Città della Salute e della Scienza University Hospital, Torino, Italy; 9 Department of Human Genetics, Radboud University Medical Center, Nijmegen, the Netherlands; 10 Department of Clinical Genetics and Genomics, Karolinska University Hospital, Department of Molecular Medicine, Karolinska Undiagnosed Disease Program, Karolinska Institutet, Stockholm, Sweden; 11 Department of Pediatric Neurology/Emma Children’s Hospital, Amsterdam UMC, Amsterdam Leukodystrophy Center, University of Amsterdam, Amsterdam, the Netherlands; 12 Department of Genetics, University of Groningen, University Medical Center Groningen, Groningen, the Netherlands; 13 Department of Genetics, University Medical Center Utrecht, Utrecht, the Netherlands; 14 Department of Clinical Genetics, Erasmus MC University Medical Center Rotterdam, Rotterdam, the Netherlands; 15 Department of Pediatric Neurology, Fukuoka Children’s Hospital, Fukuoka, Japan; 16 Department of Pediatrics, Showa University School of Medicine, Tokyo, Japan; 17 Assistance Publique-Hopitaux de Paris, Sorbonne Université, Departement de Génétique, Groupe Hospitalier Pitie-Salpetriere et Hopital Trousseau, Paris, France; 18 Genetics Department, Hospital Kuala Lumpur, Kuala Lumpur, Malaysia; 19 Department of Human Genetics, Yokohama City University Graduate School of Medicine, Yokohama, Japan; 20 Department of Public Health and Pediatrics, Pediatric Clinical Genetics, Regina Margherita Children’s Hospital, University of Turin, Turin, Italy; 21 Department of Clinical Genetics, Helsinki University Hospital, Helenski, Finland; 22 Department of Clinical Genetics and Genomics, Sahlgrenska University Hospital, Department of Biomedicine, Sahlgrenska Academy, Gothenburg University, Gothenburg, Sweden; 23 Amsterdam UMC, Emma Center for Personalized Medicine, Amsterdam, the Netherlands

**Keywords:** *CUL3*, intellectual disability, DNA methylation, episignature, NEDAUS, genotype-phenotype correlation

## Abstract

Neurodevelopmental disorder with or without autism or seizures (NEDAUS) is a neurodevelopmental disorder characterized by global developmental delay, speech delay, seizures, autistic features, and/or behavior abnormalities. It is caused by *CUL3* (Cullin-3 ubiquitin ligase) haploinsufficiency. We collected clinical and molecular data from 26 individuals carrying pathogenic variants and variants of uncertain significance (VUS) in the *CUL3* gene, including 20 previously unreported cases. By comparing their DNA methylation (DNAm) classifiers with those of healthy controls and other neurodevelopmental disorders characterized by established episignatures, we aimed to create a diagnostic biomarker (episignature) and gain more knowledge of the molecular pathophysiology. We discovered a sensitive and specific DNAm episignature for patients with pathogenic variants in *CUL3* and utilized it to reclassify patients carrying a VUS in the *CUL3* gene. Comparative epigenomic analysis revealed similarities between NEDAUS and several other rare genetic neurodevelopmental disorders with previously identified episignatures, highlighting the broader implication of our findings. In addition, we performed genotype-phenotype correlation studies to explain the variety in clinical presentation between the cases. We discovered a highly accurate DNAm episignature serving as a robust diagnostic biomarker for NEDAUS. Furthermore, we broadened the phenotypic spectrum by identifying 20 new individuals and confirming five previously reported cases of NEDAUS.

## Teaser

In this study, we identify a novel DNA methylation episignature as a sensitive diagnostic biomarker for NEDAUS, a neurodevelopmental disorder linked to CUL3 haploinsufficiency. By analyzing data from 26 individuals, we reveal overlaps with other disorders and broaden the phenotypic spectrum of NEDAUS through genotype-phenotype correlations.

## Introduction

*CUL3* (Cullin-3 ubiquitin ligase; OMIM: 603136) haploinsufficiency is associated with a neurodevelopmental disorder (NDD) with or without autism or seizures (NEDAUS; OMIM: 619239). This NDD is characterized by motor and intellectual developmental delay, speech delay, seizures, autistic features, and/or behavior abnormalities.^1^^,^^2^^,^^3^^,^^4^

*CUL3* is located at chromosome 2q36.2 and is a critical component of the Cullin-RING E3 ubiquitin ligase complex. This protein complex is involved in the regulated degradation of specific target proteins within the cell through the ubiquitination pathway. CUL3 acts as a scaffold protein, orchestrating the assembly of the complex and facilitating the ubiquitination of various substrates.^5^^,^^6^

The ubiquitination pathway is a fundamental cellular process that controls protein levels and functions. It involves attaching small protein tags, called ubiquitin to target proteins, which can mark them for degradation, alter their activity, or influence their location. This pathway relies on enzymes called E1, E2, and E3 to add ubiquitin to target proteins and deubiquitinases to remove ubiquitin when necessary. By modulating the ubiquitination of specific proteins, cells can precisely regulate various vital processes such as protein turnover, signal transduction, DNA repair, and cell-cycle progression. Previously, we successfully demonstrated two unique DNA methylation (DNAm) episignatures of genes that are connected to the ubiquitination pathway, *TRIP12* and *USP7*.^7^^,^^8^ TRIP12 functions as an E3 ligase that can target specific proteins for ubiquitination. USP7 can deubiquitinate proteins, preventing their degradation.^9^ CUL3 acts as a scaffold to facilitate the ubiquitination process, potentially interacting with E3 ligases like TRIP12.^6^ Together, CUL3, TRIP12, and USP7 proteins contribute to a regulatory network that fine-tunes the levels of key proteins within the cell and controls various cellular processes. Based on those functions and connections, we hypothesize that pathogenic *CUL3* variants may represent a unique episignature.^10^ Such DNAm signatures offer valuable applications in the field of diagnostic analysis and interpretation of variants of uncertain significance (VUS) in these genes.^11^^,^^12^ Currently, there are more than 100 episignatures that have been published and implemented in clinical diagnostics through EpiSign classifier technology.^13^

In this study, we aimed to (1) provide further insights into the clinical manifestation and molecular spectrum of individuals with NEDAUS, (2) create and validate a DNAm episignature for *CUL3* of individuals with pathogenic *CUL3* variants, and (3) analyze the similarities of global DNAm classifiers between NEDAUS and other NDDs with previously established unique episignatures.

## Materials and methods

### Ethics declaration

The study was conducted in accordance with the regulations of the Western University Research Ethics Board (REB116108 and REB106302) and the Medical Ethical Committee of the Amsterdam University Medical Center (Amsterdam, the Netherlands). We obtained written informed consent from the participants or their substitute decision maker to publish patients’ clinical and genetic information.

### Subjects and study cohort

In this study, we examined 26 individuals (15 males and 11 females), 5 of which were described before.^3^^,^^14^ The identification of *CUL3* variants was carried out in a clinical setting using exome sequencing (ES), genome sequencing, and chromosomal microarray. Gene variants were classified according to the guidelines of the American College of Medical Genetics and Genomics (ACMG) and the Association for Molecular Pathology.^15^^,^^16^ We assembled a cohort of individuals in whom diagnostic investigations had identified a *CUL3* variant; eight had a nonsense *CUL3* variant, five had a missense *CUL3* variant, nine had a frameshift *CUL3* variant, three had a splice site *CUL3* variant, and one particpant had a large deletion encompassing multiple genes, including *CUL3*.

We obtained written informed consent from the participants or their legal guardian to publish patients’ clinical and genetic information. Explicit consent for the publication of individual’s photographs was obtained separately. The cases were divided into two distinct cohorts, one for discovering the episignature (*n* = 17) and another for validation and assessment (*n* = 9). The discovery cohort was utilized for probe selection and constructing the classification model.

Several *in silico* predictors were used for analysis of pathogenicity, including PolyPhen-2 HumVar, CADD, Align GVGD, SIFT, and MutationTaster2021.^17^^,^^18^^,^^19^^,^^20^^,^^21^

### Methylation analysis

DNA extraction from peripheral blood followed standard procedures. Subsequently, DNA methylation profiling was conducted on the extracted samples using Illumina Infinium methylation EPIC bead chip arrays (San Diego, CA) according to the manufacturer’s guidelines. The analyses of episignatures were based on our laboratory’s previously published protocols.^13^^,^^22^ In summary, the intensity data files containing signals for methylated and unmethylated DNA were subjected to analysis in R (version 4.1.1). Principal-component analysis was carried out each time to scrutinize batch structure and detect any outliers among the cases or controls. To normalize the methylation data, we applied the Illumina normalization method with background correction, employing the minfi package (version 1.40.0) in R.^23^ Probes meeting the following criteria were excluded: probes with a detection *p* value exceeding 0.1, probes located on chromosomes X and Y, probes containing SNPs in or near the CpG interrogation or single-nucleotide extension sites, and probes exhibiting cross-reactivity with other genomic regions.^13^ Additionally, we removed probes with beta values of 0 and 1, as well as the top 1% most variable probes. DNA methylation assessment was performed as follows: twice for episignature detection in biomarker discovery and once to evaluate the global *CUL3* DNA methylation classifier, as described in the results section. For matching controls, a random selection was made from the EpiSign Knowledge Database (EKD),^24^ with matching based on age, sex, and array type using the MatchIt package (version 4.3.4).^25^ The standard procedure for entering samples or cases involves requesting accompanying controls from the same batch. This practice enables us to effectively manage and account for potential batch-related effects. Methylation levels for each probe, represented as beta values, were transformed into M values through logit transformation. Subsequently, linear regression analysis was employed (limma package, version 3.50.0) to pinpoint differentially methylated probes (DMPs).^26^ Estimated blood cell proportions were incorporated into the model matrix as confounding variables using the algorithm developed by Houseman et al*.*^27^ *p* values were adjusted using the eBayes function in the limma package.^26^

### Probe selection and training of the machine classifier

The selection of probes for both the initial discovery and the final episignatures occurred in multiple sequential steps. Initially, we retained a subset of probes based on the highest product of the absolute methylation differences between cases and controls and the negative logarithm of *p* values. Following this, we conducted a receiver operating characteristic (ROC) curve analysis, resulting in the retention of probes with the highest area under the ROC curve values. Probes with pairwise correlations exceeding certain thresholds, calculated using Pearson’s correlation coefficients for all probes, were subsequently removed. To further evaluate the selected probes, we applied unsupervised clustering models, including hierarchical clustering (heatmap) using Ward’s method based on Euclidean distance, utilizing the ggplot2 package in R (version 3.1.1), and multidimensional scaling (MDS) by scaling the pairwise Euclidean distances between samples. To assess the robustness of the episignatures, we conducted 18 rounds of leave-one-out cross-validation. In each round, one case sample was designated as the testing sample, while the remaining samples were used for probe selection. We visualized the corresponding unsupervised clustering plots. For the construction of a binary prediction model, we utilized the e1071 R package (version 1.7–9) to train a support vector machine (SVM) classifier, following the previously described methodology.^13^^,^^22^

### Functional annotation and comparison between EpiSign version 5 classifier cohorts

Functional annotation and comparisons with the EpiSign cohort were conducted following our previously documented methods.^22^ In summary, we generated heatmaps and Circos plots to assess the proportion of DMPs shared between the *CUL3* episignature and the other 99 neurodevelopmental conditions within the EpiSign clinical classifier.

Heatmaps were generated using the R package pheatmap (version 1.0.12), while Circos plots were created using the R package circlize (version 0.4.15).^28^ To explore relationships across all NDD cohorts without introducing bias based on the number of selected DMPs, we performed clustering analysis on the top *N* DMPs for each cohort. These DMPs were selected based on their *p* values, with the top 500 DMPs chosen for each cohort. The resulting distances and similarities between cohorts were visualized using a tree-and-leaf plot. The tree-and-leaf plots, generated using the R package TreeAndLeaf (version 1.6.1), also provided additional information such as the global mean methylation difference and the total number of DMPs identified for each cohort. To determine the genomic locations of the DMPs, we annotated the probes with respect to CpG islands (CGIs) and genes using the R package annotatr (version 1.20.0),^29^ in conjunction with AnnotationHub (version 3.2.2) and specific annotations (hg19_cpgs, hg19_basicgenes, hg19_genes_intergenic, and hg19_genes_intronexonboundaries). CGI annotations encompassed CGI shores (0–2 kb on both sides of CGIs), CGI shelves (2–4 kb on both sides of CGIs), and inter-CGI regions encompassing all remaining areas. For gene annotations, “promoters” included regions up to 1 kb upstream of the transcription start site (TSS), while “promoter+” referred to the region 1–5 kb upstream of the TSS. Annotations related to untranslated regions (5′ UTR and 3′ UTR), exons, introns, and exon/intron boundaries were consolidated into the “gene body” category. A chi-squared goodness of fit test was carried out in R to assess the significance of differences between the background DMP annotation distribution and the annotation distribution within the NEDAUS cohort. *p* values were calculated for both annotation categories, namely genes and CGIs.

## Results

### Molecular characterization of the *CUL3* cohort

**Figure 1:**
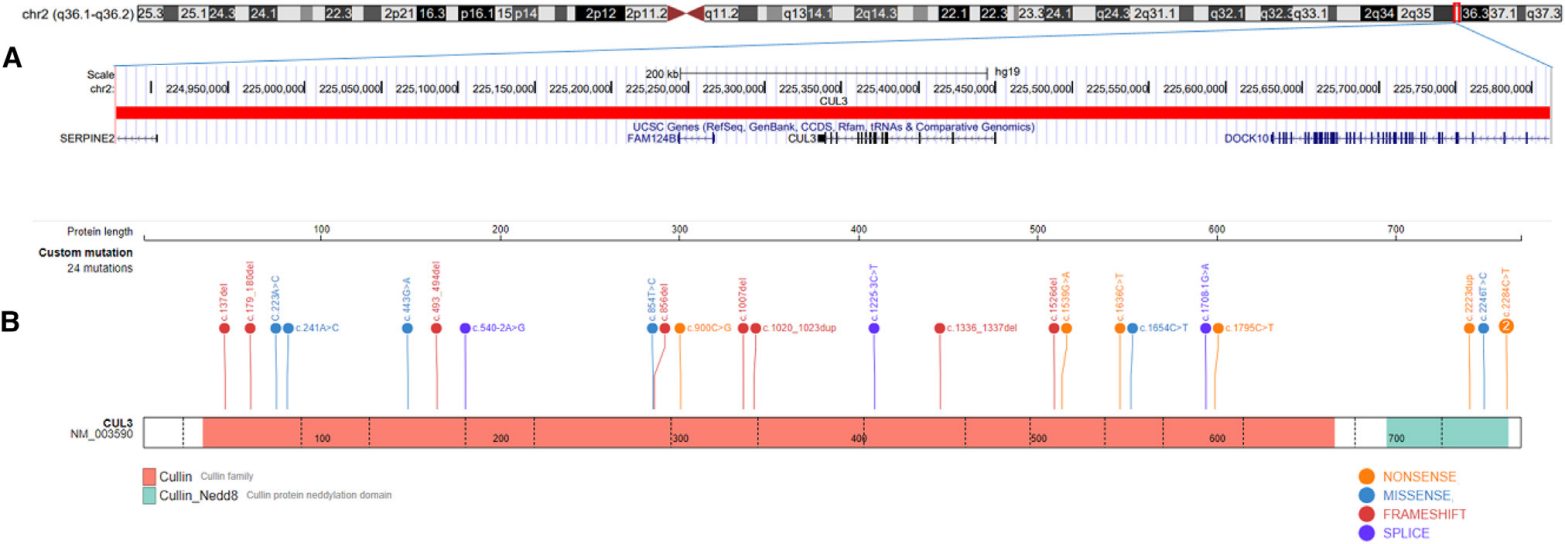
Overview of our CUL3 cohort Cytogenetics band and known genes are represented in this figure using the UCSC genome browser 2009 (GRCh37/hg19) genome build. (A) Deletion of chromosome 2q36.1q36.2 in case 18 is represented by the horizontal red bar and the genes contained within the region listed below. (B) Overview of other CUL3 variants in this cohort. Image is edited from St. Jude Cloud protein paint image (https://pecan.stjude.cloud/proteinpaint).

The molecular details of our cohort are summarized in Table 1 and Figure 1. All individuals carried an *CUL3* variant, and one participant had a large CNV including *CUL3.* The comprehensive findings from the *in silico* analyses are provided in Table S1.

**Table 1:** *CUL3* variants detected in participants 1–26 NM_003590.5

Case	Label	Gender	Variant	Variant type	Classification	Inheritance
1	NEDAUS	M	c.900C>G p.(Tyr300∗)	nonsense	LP	*de novo*
2	NEDAUS	M	c.137delG p.(Arg46Leufs∗32)	frameshift	LP	*de novo*
3	NEDAUS	F	2266C>T p.(Arg756∗)	nonsense	LP	paternal
4	NEDAUS	M	c.1636C>T p.(Arg546∗)	nonsense	LP	NA
5	NEDAUS	M	c.1336_1337del p.(Val446Phefs∗2)	frameshift	LP	*de novo*
6	NEDAUS	M	c.2223dup p.(Lys742∗)	nonsense	LP	NA
7	NEDAUS	F	c.1539G>A p.(Trp513∗)	nonsense	P	*de novo*
8	NEDAUS	M	c.179_180delAT p.(Asn60Serfs∗8)	frameshift	LP	paternal
9	NEDAUS	M	c.1007del p.(Lys336Argfs∗13)	frameshift	LP	*de novo*
10	NEDAUS	F	c.2246T>C p.(Ile749Thr)	missense	LP	*de novo*
11	NEDAUS	M	2266C>T p.(Arg756∗)	nonsense	LP	paternal
12	NEDAUS	M	c.1020_1023dup p.(Ile342leufs∗11)	frameshift	LP	*de novo*
13	NEDAUS	M	c.540-2A>G p.?	splice site	LP	*de novo*
14	NEDAUS	M	c.1654C>T p.(Arg552∗)	nonsense	P	*de novo*
15	NEDAUS	F	c.1777C>T p.(Gln593∗)	nonsense	LP	*de novo*
16	NEDAUS	F	c.493_494del p.(Leu165Ilefs∗37)	frameshift	LP	*de novo*
17	NEDAUS	M	c.978del p.(Ala327Leufs∗22)	frameshift	LP	*de novo*
18	NEDAUS_VUS_positive	M	arr[GRCh37] 2q36.1q36.2(224877730_225811469)x1	deletion	VUS	NA
19	NEDAUS_VUS	F	c.854T>C p.(Val285Ala)	missense	VUS	*de novo*
20	NEDAUS_VUS	M	C.223A>C p.(Thr75Pro)	missense	VUS	paternal
21	NEDAUS_VUS	F	c.443G>A p.(Arg148Gln)	missense	VUS	*de novo*
22	NEDAUS_VUS	F	c.223A>C p.(Thr75Pro)	missense	VUS	*de novo*
23	NEDAUS_negative	M	c.1207-3C>TC>T p.?	splice site	LP	*de novo*
24	NEDAUS_negative	F	c.1708-1G>A p.?	splice site	LP	*de novo*
25	NEDAUS_negative	F	c.1526del p.(Thr509fs)	frameshift	LP	*de novo*
26	NEDAUS_negative	F	c.856delC p.(His286Ilefs∗3)	frameshift	LP	*de novo*

Participants 3 and 11 are related (father and daughter). F, female; LP, likely pathogenic; M, male; NA, not available; P, pathogenic; VUS, variants of uncertain significance.

### Clinical characterization of the *CUL3* cohort

**Figure 2:**
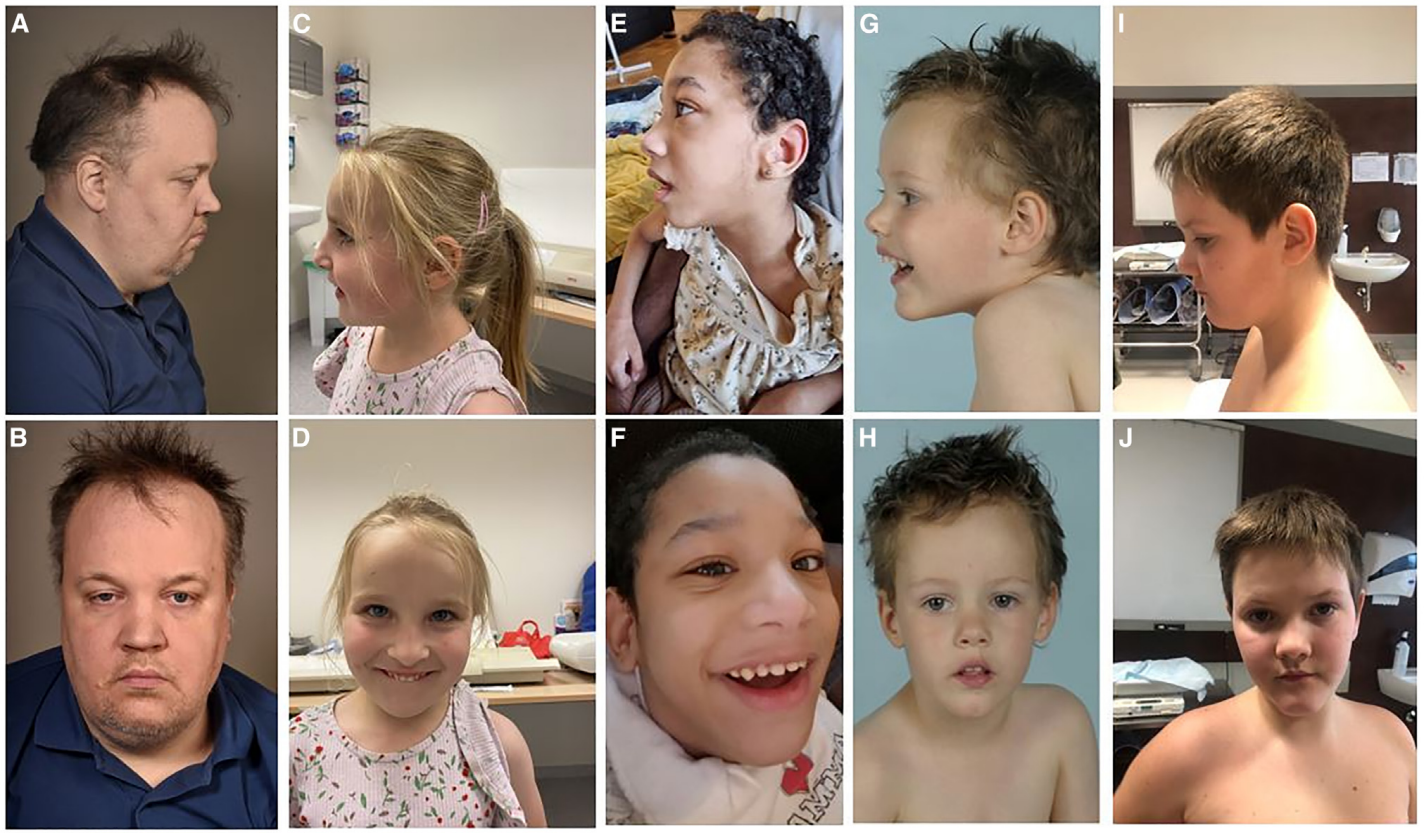
Facial features of our NEDAUS cases (A and B) Participant 4. (C and D) Participant 7. (E and F) Participant 10. (G and H) Participant 12. (I and J) Participant 17.

The clinical details of our cohort are summarized in Table S2 and Figure 2. More detailed information for each patient are available in the Table S3. The predominant characteristics observed among the total cohort were learning disorders (89%), developmental delays including speech and motor skills (88%), behavioral abnormalities (76%), intellectual disabilities (IDs; 77%), and food-hand abnormalities (60%). The most frequent facial features were a large forehead (52%) and a pointed chin (50%). However, no typical recognizable facial features associated with this syndrome were noticed—in other words, the observed facial features in patients were typically mild and heterogeneous.

### Identification of the NEDAUS episignature

The DNAm episignature discovery cohort probe set effectively distinguished between cases and controls. However, four participants (samples 23–26) did not align with the episignature and were removed from the training set. These samples clustered with controls in both heatmap and MDS plot and had methylation variant pathogenicity (MVP) scores near 0. All the other cases (1–17) clustered away from controls in both heatmap and MDS plot and had MVP scores near 1. Next, using multiple rounds of leave-one-out cross-validation, we observed a robust episignature, which was visualized with unsupervised clustering methods, such as heatmap and MDS plots (Figure S1).

### Assessment of the NEDAUS episignature

**Figure 3:**
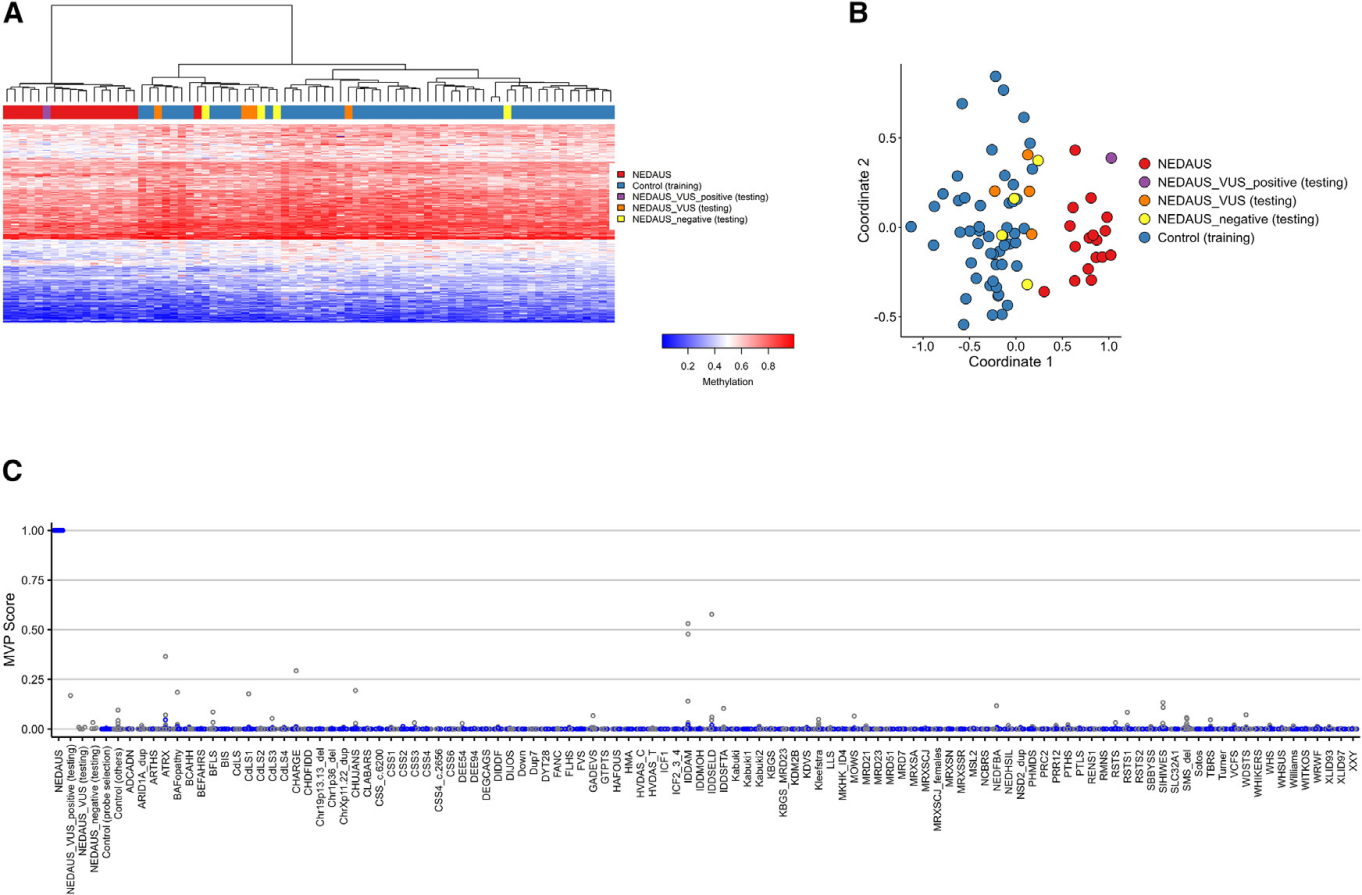
Assessment of the NEDAUS episignature (A) The Euclidean hierarchical clustering heatmap shows each column as an NEDAUS discovery case (highlighted in red), along with negative samples (in purple), VUS (in orange), and a VUS^+^ sample (in yellow). Each row represents a specific probe chosen for this episignature. A clear distinction is evident between the cases (in red) and controls (in blue). (B) The multidimensional scaling (MDS) plot displays the separation between NEDAUS cases and controls, including the negative sample identified in (A). (C) In the SVM classifier model, the selected NEDAUS episignature probes were used for training. Seventy-five percent of controls and 75% of samples from other neurodevelopmental disorders (NDDs; shown in blue) were used for training, while the remaining 25% of controls and 25% of the other disorder samples (gray) were used for testing. The plot indicates that all NEDAUS samples have MVP scores near 1. Additionally, NEDAUS testing samples, including negatives and VUS, generally showed low MVP scores, except for a VUS sample that scored 0.16.

Previously, it was shown that episignatures can be used to reclassify VUS effectively.^11^^,^^30^ Therefore, we utilized the NEDAUS episignature to test five VUS samples (18–22). The analysis revealed that only one VUS sample (18) aligned with the NEDAUS episignature. The remaining VUS samples (19–22) did not match the NEDAUS episignature (Figure 3).

Subsequently, we added the positive VUS to the training cohort (Figure S2) and we re-performed leave-one-out cross-validation to evaluate the reproducibility and sensitivity of our final episignature. Unsupervised hierarchical and MDS clustering methods showed that each testing sample correctly clustered with the training cases in every round of cross-validation (Figure S3). The binary SVM model was constructed using the 213 differentially methylated probes (Table S4).

### Functional annotation and comparison between EpiSign version 5 classifier cohorts

**Figure 4:**
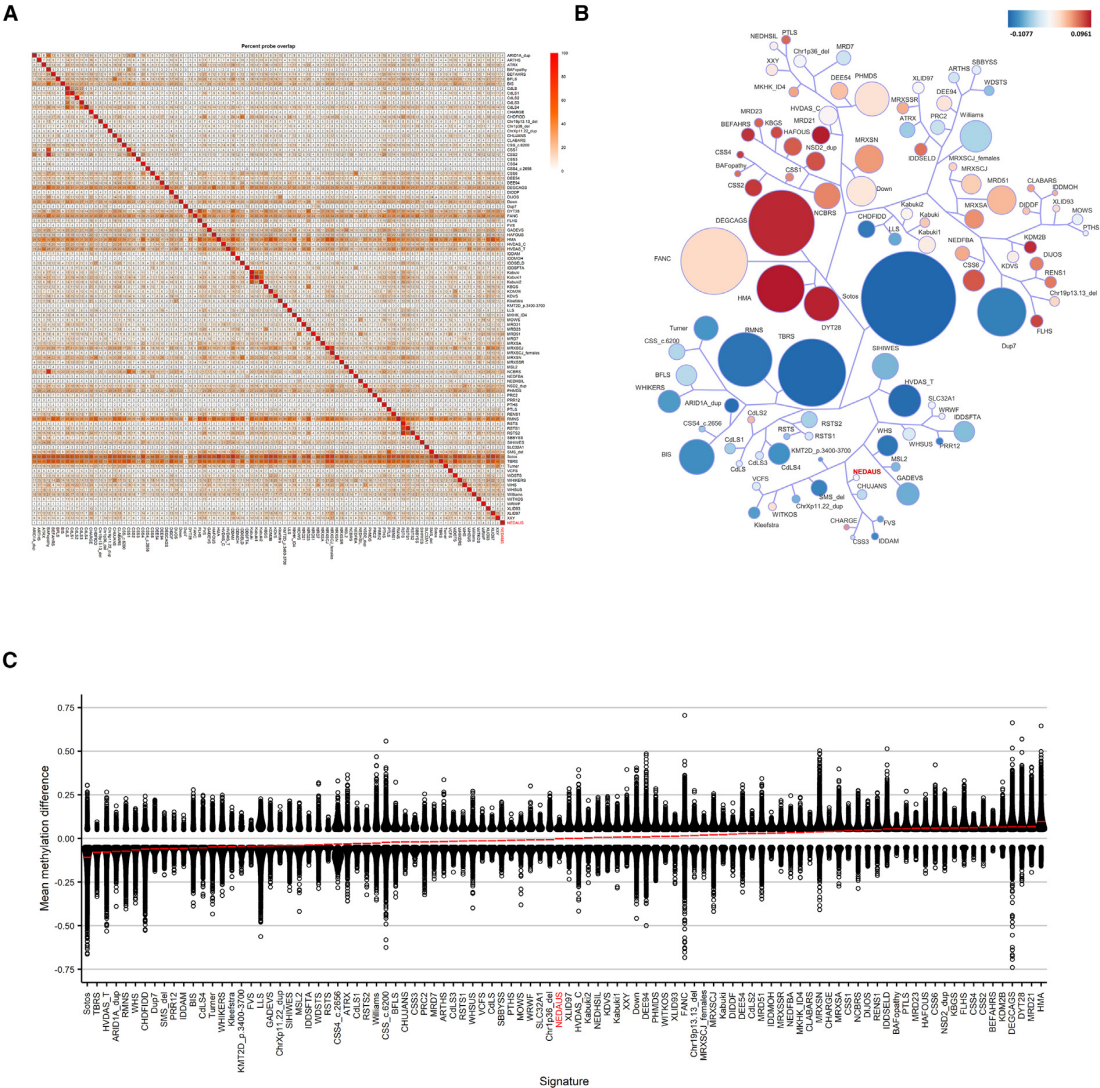
DMPs shared between the NEDAUS cohort and other EpiSign established episignatures (A) Methylation probe overlap, showing the percentage of DMPs shared between disorders on a color scale from white (0%) to red (100%). Each square in the graph indicates the percentage of common probes between two syndromes, with the percentage of DMPs from the syndrome on the bottom bar that also appears in the DMPs of the syndrome on the right-hand side bar. (B) A tree-and-leaf diagram represents each cohort as a node. Syndromes with more similar methylation levels are closer on the tree. Node size corresponds to the ratio of the number of DMPs to the total number of probes, while node color reflects the overall mean methylation difference in the corresponding cohort. (C) Comparison of global mean methylation differences between syndromes with known episignatures, highlighting the overall hypomethylation classifier of the NEDAUS episignature.

The DMPs were identified by comparing the NEDAUS-affected samples with matched controls from the EKD. We mapped the number of shared DMPs between NEDAUS and the 99 other EpiSign disorders.^22^ The NEDAUS cohort had the highest percentage of DMP overlap with intellectual developmental disorder, autosomal dominant 51 (MRD51; *KMT5B*) (20%). We also evaluated the mean beta value differences between NEDAUS and the other disorders with a known EpiSign signature. Predominant hypomethylation changes were observed for the NEDAUS cohort. The tree-and-leaf plot clustering analysis revealed similarities between NEDAUS and Chung-Jansen syndrome (CHUJANS; *PHIP*) (Figure 4).

The genomic locations of DMPs were annotated in relation to genomic features. In our analysis, 49% of the DMPs were located in the inter-CGI region, 22% in shelves, 21% in islands, and only 7% in shores. We also detected a significant (*p* < 1.15e−12) enrichment of DMPs in gene coding regions—39% (Figure S4). Notably, no differential methylated regions (DMRs), defined as at least five consecutive significantly differentially methylated DMPs within 1 kb, were identified.

### Phenotype comparison between positive and negative cases

Next, we compared phenotypic characteristics between DNAm signature-positive (*n* = 18) and signature-negative (*n* = 8) cases (Table S2). We found that intrauterine growth restriction (IUGR) was present in only 29% of the signature-negative cohort compared to the 59% in the signature-positive cohort, and feeding problems were present in 36% of the signature positives but only present in one individual (14%) in the signature negatives. Similarly, attention-deficit/hyperactivity disorder (ADHD) was observed in 33% of the signature positives but not observed (0%) in the signature negatives. Finally, MRI abnormalities and cardiac abnormalities (mostly atrial septal defect and ventricular septal defect) were frequently noticed in the signature positives (56% and 35%, respectively) but not present in the signature negatives (0%).

## Discussion

Our study adds to the increasing evidence that DNAm patterns serve as biomarkers for Mendelian NDDs, focusing on cases with pathogenic *CUL3* variants. DNAm patterns, established early in embryonic development and detectable across all tissues, offer a promising avenue for clinical diagnostics.^30^ Identifying and validating specific DNAm episignatures for NEDAUS helps diagnose unresolved cases of ID and reclassify VUS.^31^ In this study, we aimed to characterize and validate a DNAm episignature for affected individuals carrying pathogenic *CUL3* variants, analyze the associations between phenotypic characteristics and the episignature, compare global DNAm classifiers between *CUL3* and other NDDs, and provide additional insights into the clinical features.

Our findings revealed a distinct DNAm episignature associated with likely pathogenic/pathogenic *CUL3* variants, indicating a molecular diagnostic tool for NEDAUS. By analyzing DNAm classifiers from the peripheral blood of affected individuals, we established a robust classification model. The model successfully differentiated affected individuals from unaffected controls, demonstrating the specificity and sensitivity of the NEDAUS episignature. Importantly, our SVM model confirmed the strength of selected episignature probes as biomarkers for the molecular diagnosis of this disorder. Notably, while the NEDAUS episignature is defined on relatively small effect sizes of the individual probes within the classifier, we have shown that it remains highly effective in detecting pathogenic variants. This subtle, yet distinctive, episignature underscores the precision of our diagnostic approach. Furthermore, our study highlights the specificity of the NEDAUS episignature, enabling one to distinguish from other NDDs accurately. Altogether, the high sensitivity and specificity of the NEDAUS signature allows implementation within the EpiSign diagnostic panel, and thus further contributes to this powerful diagnostic tool.

As episignatures play a pivotal role in the reclassification of VUS, we tested the applicability of our NEDAUS episignature on five affected individuals. Number 18, characterized by a large deletion including *CUL3*, clustered together within the *CUL3* cases in the MDS plot and showed an MVP score of nearly 1, indicating the presence of a positive NEDAUS episignature. This deletion was classified previously as a VUS due to uncertainties regarding the gene function. A re-evaluation of this deletion to date would have indicated that this deletion leads to the haploinsufficiency of CUL3 function and is therefore pathogenic. Moreover, this deletion additionally involved two other genes (*DOCK10*, *FAM124B*), both of which have not been associated previously with any condition. Episignatures specific to genes, like the one found in NEDAUS, can help pinpoint the specific gene or specific genetic boundaries within the CNV that are likely responsible for the DNAm aberrations. Our result indicate that this case represents a similar DNAm classifier as SNV variants in *CUL3*.^32^ The other four affected individuals carrying a VUS in *CUL3* clustered together with controls and yielded MVP scores near 0, indicating the absence of the NEDAUS episignature. Those four cases are missense variants and according to *in silico* analyses are probably not causing structural damage (Table S1). However, pathogenicity cannot be excluded.

The DNA profiles of cases 23–26 closely resembled those of controls, suggesting the absence of an NEDAUS episignature. Based on ACMG guidelines, these variants were classified as likely pathogenic.

Further *in silico* analysis of the genetic variants in samples 23 and 24 revealed notable differences. In sample 23, the variant (c.1207-3C>T) had no discernible effect on the canonical splice site. Conversely, in sample 24, the variant (c.1708-1G>A) significantly impacted the canonical splice site, yet it was unclear why this information did not align with the clustering observed in other cases. Two other individuals with frameshift variants (25 and 26) also did not cluster with the positive cases. No other explanatory variants were identified during ES. Clinically, participants 23–26 presented with milder phenotypes compared to the positive cohort, lacking features such as IUGR, MRI abnormalities, cardiac defects, and feeding difficulties. Importantly, no consistent facial dysmorphology was observed, which aligns with the broader finding that NEDAUS does not present with a characteristic facial phenotype. This clinical variability may explain why these cases did not exhibit the episignature seen in more typical NEDAUS cases. It is important to note that these cases did not present with all features commonly observed in the positive cohort, such as IUGR, MRI findings, cardiac abnormalities, and feeding problems.

We examined the DNAm patterns of our NEDAUS cohort and compared them to 99 previously identified EpiSign (version 5) disorders. We found significant overlap between NEDAUS and MRD51 DNA methylation signature. MRD51, linked to pathogenic variants in the *KMT5B* gene, encodes a histone methyltransferase, which is involved in chromatin remodeling and gene regulation.^33^ Analyzing the directionality of methylation changes in NEDAUS and comparing them with other hypomethylated cohorts, we found that NEDAUS clustered closely with CHUJANS (caused by pathogenic variants in *PHIP*).^34^ Both *PHIP* and *CUL3* are involved in the ubiquitination pathway with different functions, but they can interact with each other.^35^ Also, *CUL3* and *PHIP* are both genes known to be involved in autism spectrum disorders.^36^ These disorders show overlapping phenotypes, including ID, behavioral abnormalities, developmental delay, and speech delay. However, given their roles in neurodevelopmental pathways, there may be shared phenotypic characteristics between these two genetic conditions. Additionally, since CHUJANS is associated with obesity, further clinical research is needed to explore this feature in patients with NEDAUS. In our cohort, obesity was present in only three individuals.

In this study, we also provided clinical information of 26 individuals from 25 families with 24 different variants in *CUL3* and 1 with a CNV including *CUL3*. Out of those 26 individuals, 20 have not been reported previously.^3^^,^^14^ The main clinical findings in our study where IUGR, developmental delay including speech and motor skills, ID, behavioral abnormalities, and food-hand abnormalities (tapering/extra fingers). We have not found any evidence that typical facial features are associated with this syndrome. The evaluation of episignature-positive and -negative cases in the context of IUGUR, feeding problems, ADHD, MRI of the brain, and cardiac abnormalities (including atrial sepal defect and ventricular septal defects) indicated clear enrichments for these features within the signature positive case cohort. The presence of these specific clinical features in signature-positive cases suggests a strong phenotype-episignature association. Unlike other conditions with previously established episignatures, where the episignature may not be as tightly correlated with specific phenotypic traits, this particular association is noteworthy because it could serve as a precise diagnostic tool in clinical practice. Identifying this strong association means that the episignature can be used to reliably diagnose individuals with similar phenotypic presentations, including those syndromes where facial features may not be distinctly characteristic. This capability is particularly valuable for distinguishing conditions that share overlapping symptoms, leading to more accurate diagnoses and tailored management plans. In essence, the strong link between episignatures and specific clinical features enhances their utility as diagnostic markers, offering clearer guidance for clinical decision-making and ultimately improving patient care. Moreover, the identification of 20 new, previously unreported cases with this NDD, broadens the known clinical spectrum of NEDAUS. This significant addition of unreported cases underscores the importance of our findings, as it not only highlights new phenotypic variations but also demonstrates the episignatures’ robustness in identifying patients with CUL3-related conditions. This broader spectrum enhances genetic counseling and refines clinical strategies, ensuring better management for a wider range of affected patients.

The mechanisms through which *CUL3* mutations might lead to altered DNA methylation patterns remain an area of ongoing research. CUL3 functions as part of the Cullin-RING E3 ubiquitin ligase complex, which regulates the degradation of numerous cellular proteins, including those involved in epigenetic modifications. One possible hypothesis is that disruptions in *CUL3*-mediated ubiquitination may affect the stability and activity of chromatin-modifying enzymes, such as histone methyltransferases and DNA methyltransferases. These enzymes play a critical role in maintaining DNA methylation patterns and chromatin structure. Dysregulation of this pathway could lead to widespread epigenetic changes, including the aberrant DNA methylation observed in NEDAUS. Additionally, *CUL3* has been implicated in neurodevelopmental pathways, and disruptions in these pathways could also indirectly influence DNA methylation by altering the expression of genes involved in neurodevelopment and synaptic function. Further research is needed to elucidate the precise molecular mechanisms by which *CUL3* mutations impact DNA methylation, but our findings suggest that these epigenetic changes are integral to the pathophysiology of NEDAUS.

Overall, the findings from this study contribute to a growing body of evidence supporting the utility of DNA methylation patterns as diagnostic biomarkers in NDDs.

### Conclusion

Here, we present a precise and distinctive DNAm episignature for patients carrying pathogenic variations in *CUL3* or CNVs encompassing *CUL3*. This episignature is set to enhance the EpiSign classifier, facilitating clinical testing in individuals with suspected NEDAUS. In addition, based on the clinical phenotype of 20 new cases with NEDAUS, we further delineate the clinical spectrum for this NDD, thereby enhancing genetic counseling and refining clinical strategies for affected patients.

## Data and code availability

Raw DNA methylation data are available from the authors on reasonable request.

## Acknowledgments

We would like to thank the participants described in this study. Funding for this study was provided in part by the 10.13039/501100000023Government of Canada through 10.13039/100008762Genome Canada and the 10.13039/501100000092Ontario Genomics Institute (OGI-188). L.v.d.L. received the AR&D Travel Grant from 10.13039/100019573Amsterdam UMC, which provided financial support for this work. T.S.B. was supported by the 10.13039/501100003246Netherlands Organisation for Scientific Research (ZonMw Vidi, grant 09150172110002) and acknowledges ongoing support from EpilepsieNL and 10.13039/100002736CURE Epilepsy. N.M. and T.M. were supported by the 10.13039/100007449Takeda Science Foundation, with additional support for T.M. from 10.13039/501100001691JSPS KAKENHI under grant nos. JP23K27568 and JP23K18278.

## Author contributions

Conceptualization: L.v.d.L., A.S., K.R., M.M.A.M.M., B.S., P.H., and M.M.v.H. Data curation: L.v.d.L., A.S., K.R., S. Haghshenas, and M.A.L. Formal analysis: L.v.d.L., A.S., K.R., S. Haghshenas, and M.A.L. Investigation: L.v.d.L., L.K., P.L., Y.A., P.B., A.B., S.d.M., B.B.A.d.V., A.D.V., M.E., J.C.H., R.H., S. Hopman, S.G.K., M.K., B.K., H.Y.K., N.L.-H., S.M.M., G.M.S.M., C.M., N.M., T.M., C.M., A. Nähri, A. Nordgren, R.P., A.M.P., S.T., Y.v.B., M.J.v.d.B., J.J.v.d.S., T.S.B., M.A., M.M.v.H. Methodology: M.M.A.M.M., B.S., P.H., and M.M.v.H. Project administration: M.M.A.M.M., B.S., P.H., and M.M.v.H. Supervision: M.M.A.M.M., B.S., P.H., and M.M.v.H. Validation: L.v.d.L. and A.S. Visualization: L.v.d.L. and A.S. Writing – original draft: L.v.d.L. Writing – review & editing; all authors.

## Declaration of interests

B.S. is a shareholder in EpiSign Inc., a biotech firm involved in the commercial application of EpiSign technology.

## Supplemental information

Data S1. Tables S1–S4

Document S2. Article plus supplemental information
