# Supplementary material for: CUL3-related neurodevelopmental disorder: Clinical phenotype of 20 new individuals and identification of a potential phenotype-associated episignature
Source: HGG Adv. 2024 Nov 4;6(1):100380. doi: 10.1016/j.xhgg.2024.100380 (PMC11617854; doi:10.1016/j.xhgg.2024.100380)
Supplement: Document S1. Figures S1–S4 [file mmc1.pdf]

HGGA, Volume *6*

Supplemental information

CUL3-related neurodevelopmental disorder: Clinical phenotype of 20 new individuals and identification of a potential phenotype-associated episignature

Liselot van der Laan, Ananília Silva, Lotte Kleinendorst, Kathleen Rooney, Sadegheh Haghshenas, Peter Lauffer, Yasemin Alanay, Pratibha Bhai, Alfredo Brusco, Sonja de Munnik, Bert B.A. de Vries, Angelica Delgado Vega, Marc Engelen, Johanna C. Herkert, Ron Hochstenbach, Saskia Hopman, Sarina G. Kant, Ryutaro Kira, Mitsuhiro Kato, Boris Keren, Hester Y. Kroes, Michael A. Levy, Ngu Lock-Hock, Saskia M. Maas, Grazia M.S. Mancini, Carlo Marcelis, Naomichi Matsumoto, Takeshi Mizuguchi, Alessandro Mussa, Cyril Mignot, Anu Närhi, Ann Nordgren, Rolph Pfundt, Abeltje M. Polstra, Slavica Trajkova, Yolande van Bever, Marie José van den Boogaard, Jasper J. van der Smagt, Tahsin Stefan Barakat, Mariëlle Alders, Marcel M.A.M. Mannens, Bekim Sadikovic, Mieke M. van Haelst, and Peter Henneman

A

B

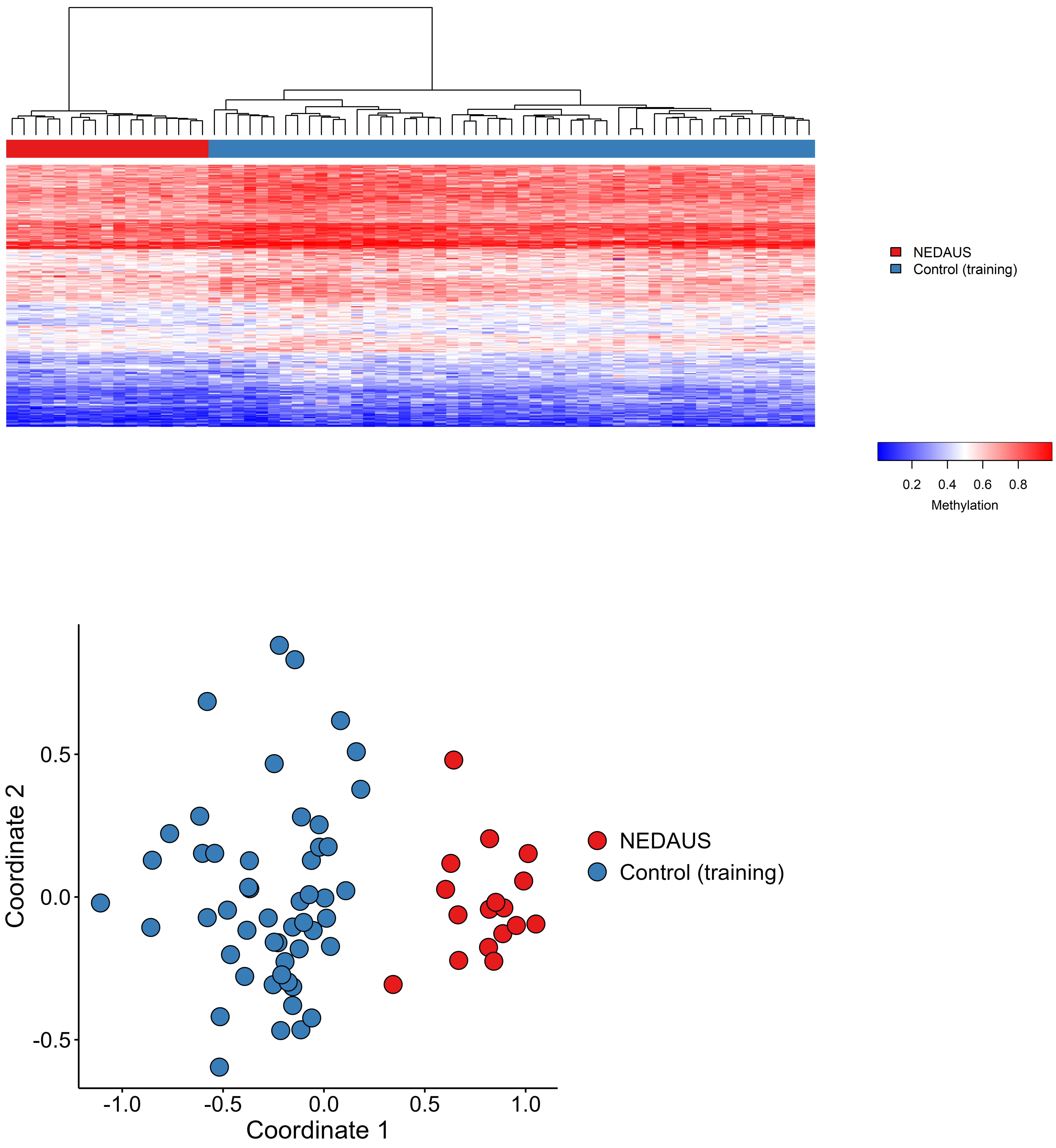

Supplementary Figure 1; NEDAUS Episignature Discovery Cohort. (A) Euclidean hierarchical clustering heatmap, where each column stands for a NEDAUS discovery case or control, and each row represents a probe chosen for the episignature. The heatmap clearly differentiates cases (in red) from controls (in blue). (B) Multidimensional scaling (MDS) plot demonstrating the separate clustering of NEDAUS cases and controls

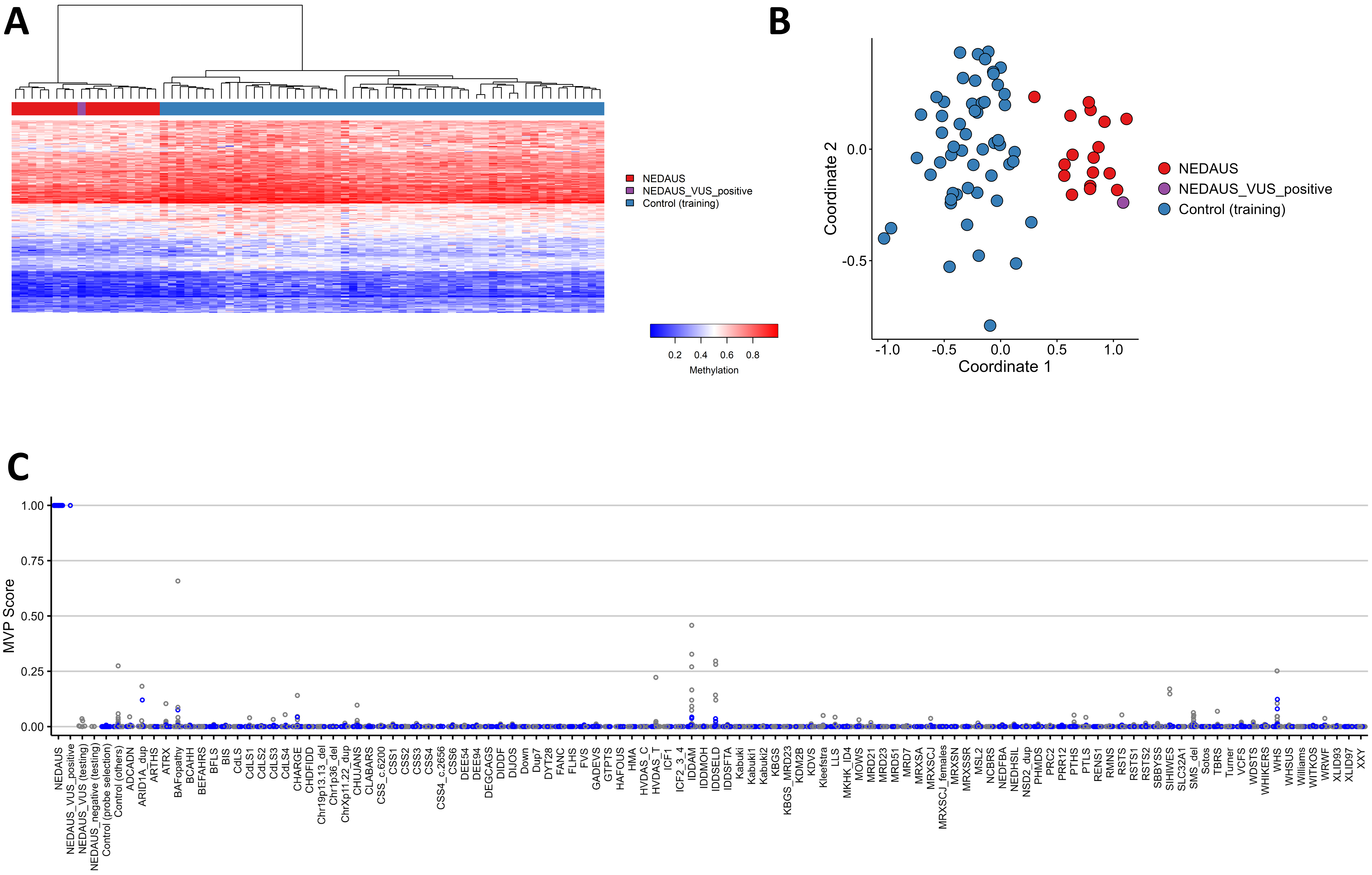

Supplementary Figure 2; NEDAUS Episignature Discovery and Positive VUS Combined Training. (A) Euclidean hierarchical clustering heatmap depicting NEDAUS cases and controls, with each column representing a case or control, and each row indicating a selected probe for the episignature. The heatmap shows a distinct separation between cases (in red and purple) and controls (in blue). (B) The MDS plot illustrates the separation of NEDAUS cases and controls, highlighting the similarity between NEDAUS pathogenic samples and the positive VUS. (C) The SVM classifier model was trained using selected NEDAUS episignature probes, 75% of controls, and 75% of other neurodevelopmental disorder samples (in blue). The remaining 25% of controls and 25% of other disorder samples (in grey) were reserved for testing. The plot indicates that NEDAUS training samples had MVP scores near 1.

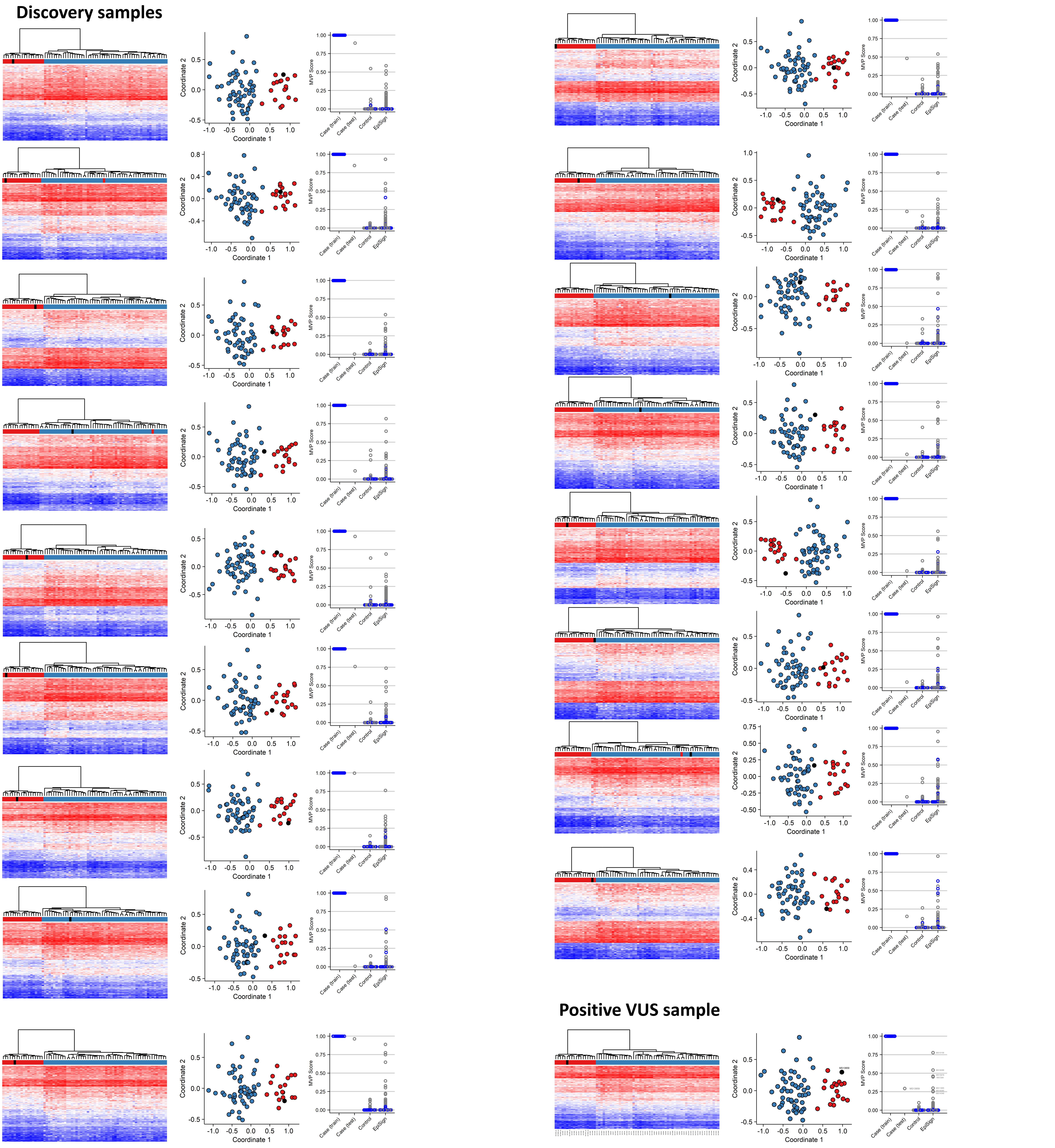

Supplementary Figure 3; Combined Discovery and Positive VUS Cohort Leave-25%-Out Cross Validation. This figure illustrates eighteen rounds of leave-25%-out cross-validation. In each round, test case samples (in black) are used for testing, while the remaining cases (in red) are used for episignature training, along with control training samples (in blue) in both the heatmap and MDS plots. The final plots show the MVP scores of the SVM classifier model, which is trained using the selected NEDAUS episignature probes from training cases, 75% of controls, and other EpiSign samples (in blue). The remaining 25% of controls and other disorder samples are used for testing, along with the NEDAUS testing case samples (in grey).

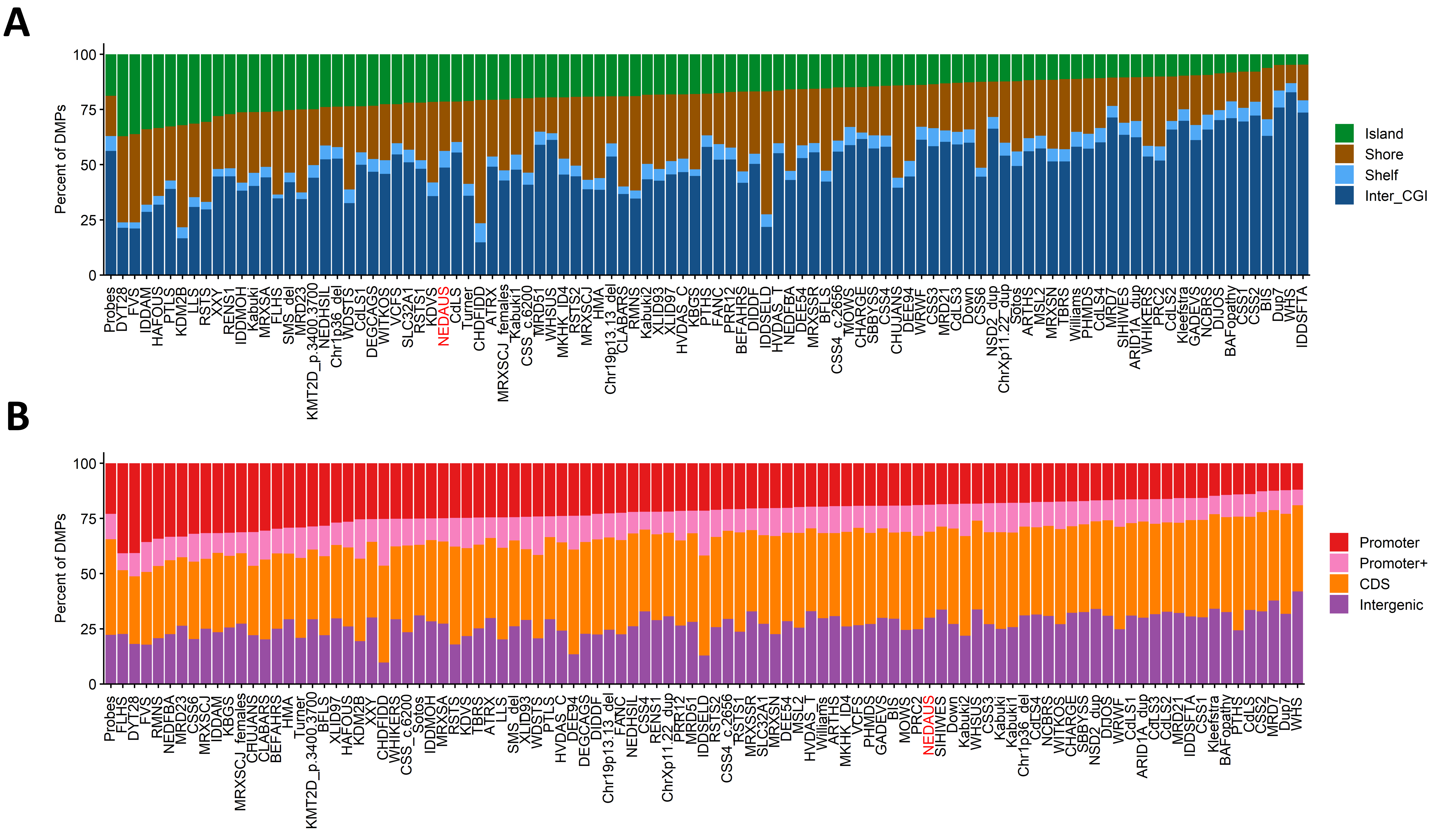

Supplementary Figure 4. DMPs annotated in the context of CpG islands and genes . (A) DMPs in relation to genes: Promoter (0-1 kb upstream of the transcription start site), Promoter+ (1-5 kb upstream of the TSS), CDS (coding sequence), and Intergenic (other genome regions). (B) DMPs in CpG islands: Island (CpG islands), Shore (within 0-2 kb of a CpG island boundary), Shelf (within 2-4 kb of a CpG island boundary), and Inter_CGI (other genome regions).
